# Pervasive systemic disparities: medical-related gaslighting, trauma, and distrust among LGBTQ+ people in the United States

**DOI:** 10.3389/fsoc.2025.1488888

**Published:** 2025-02-12

**Authors:** Dustin Z. Nowaskie, Dehandra Blackwood, Frank Garcia

**Affiliations:** ^1^Department of Psychiatry and the Behavioral Sciences, Keck School of Medicine, University of Southern California, Los Angeles, CA, United States; ^2^Mailman School of Public Health, Columbia University, New York, NY, United States; ^3^Columbia University, New York, NY, United States

**Keywords:** gaslighting, LGBTQ+, pharma, provider, trauma, trust

## Abstract

**Introduction:**

Historically, LGBTQ+ people have and continue to endure discrimination across many contexts, including healthcare. Research and data from nationwide samples in the United States regarding medical-related stigma experienced by LGBTQ+ people are scant.

**Methods:**

A cross-sectional survey was distributed online to a national sample of United States residents. Participants answered questions about their healthcare, including experiences with medical-related gaslighting, trauma, and trust.

**Results:**

Compared to cisgender and heterosexual people (*n* = 857), LGBTQ+ people (*n* = 815) reported significantly higher rates of medical-related gaslighting (46.5% vs. 26.5%, *ORs* 1.75–2.80) and trauma (18% vs. 8.9%, *ORs* 1.63–2.66). Likewise, LGBTQ+ people conveyed significantly less trust (*ORs* 0.46–0.53) in primary care providers (59.8% vs. 74.1%), medical specialists (56.5% vs. 71.7%), pharmaceutical companies (17% vs. 28%), insurance companies (15.9% vs. 29.3%), and U.S. healthcare systems (17.8% vs. 30.4%).

**Discussion:**

Medical-related gaslighting, trauma, and distrust are pervasive systemic disparities among LGBTQ+ people. Addressing these challenges will require ongoing, lifelong motivation, dedication, and commitment for LGBTQ+ education, advocacy, and leadership to dismantle current prejudiced practices and foster more inclusive, supportive, affirming healthcare environments.

## Introduction

Medical gaslighting is a term that is used to describe when healthcare professionals minimize, dismiss, or outright ignore a patient’s concerns and symptoms (Sebring, 2021). While engaging in gaslighting, healthcare professionals may indicate that symptoms originated from various factors such as stress, anxiety, weight, and/or a person’s identity or lifestyle rather than attempting to conduct a more thorough examination. The term “gaslighting” is from a 1940 screenplay by Patrick Hamilton, which tells the story of a man who manipulates his wife into doubting her sanity, thereby gaslighting her (Klein, 2023). Since then, the term has been popularized to explain similar instances of mistreatment and neglect, often stemming from biases and prejudices. As a result, medical gaslighting functions as a method in which healthcare professionals may consciously and/or subconsciously perform implicit and explicit forms of discrimination against individuals who may already be marginalized by social, political, and medical systems.

The term “medical gaslighting” received much more attention and was widely used in connection with individuals who suffered from long coronavirus disease 19 (COVID-19) (Wise, 2022). These individuals continued to experience symptoms of the COVID-19 virus longer than what medical professionals initially assumed. Subsequently, these patients were met with doubt as a consensus was that the coronavirus should parallel influenza and subside in days. This skepticism and invalidation were notable barriers to care for these individuals, who then helped popularize the term medical gaslighting on social media to explain the injustices that they received from healthcare professionals (Wise, 2022).

Medical gaslighting echoes Miranda Fricker’s work (Fricker, 2007) on “epistemic injustice,” a term used to refer to a wrong done to someone specifically in their capacity as a knower. Fricker states there are two kinds of epistemic injustice: testimonial and hermeneutical. Testimonial injustice occurs when someone is ignored or not believed because of their sex, sexuality, gender, race, disability, etc. Meanwhile, hermeneutical injustice refers to having some significant area of one’s social experience obscured from collective understanding due to a general absence of the collective hermeneutical resource (Fricker, 2007). Examples of this injustice include the historical lack of regard for postpartum depression and sexual harassment because both concepts, until fairly recently, were not largely acknowledged or known by society. In the case of long-term COVID-19, individuals were often not believed, likely because of a general lack of understanding or recognition of what long COVID-19 truly is. Judith Butler’s theory of performativity can be used to analyze the doctor-patient relationship and power structure in medicine (Butler, 1988; Butler, 2004). In receiving a patient’s testimony, a provider performs the role of spokesperson for the institution of medicine and science, which has the privilege and power to pronounce what is real and what is not, operating from a hierarchical understanding of knowledge and perpetuating presumed norms (Sebring, 2021). Therefore, not only is the patient represented as inferior in the deceivingly mutual doctor-patient relationship (note the historical ranking of “doctor” and “patient” in this phrasing), but if the patient declares symptoms that cannot be explained by science, they must, via scientific reasoning, not be real. Medical gaslighting functions under this arrangement as it is not only influenced by societal power structures that often invalidate individuals likely due to their social positions, but it also survives due to a general lack of recognition and regard as well as novelty and operationalization of the concept and its reality.

The power structures that are performed in the doctor-patient relationship are often further influenced by a person’s social positioning in society. Many individuals who are women, people of color, gender diverse, sexually diverse, with disability, and whose bodies are otherwise perceived as abnormal or inferior under a medical gaze often lack cultural, economic, and social capital which makes them particularly at risk to many forms of injustices (Sebring, 2021). Sebring refers to these individuals as “bio-Others” and introduces this concept as an additional element to Foucault’s biopower, the mechanisms of power that govern people and populations (Foucault, 2008). Medical gaslighting is one component of biopower used to subjugate bio-Others. It disproportionately and naively places the responsibility for health issues on the individual by writing off health concerns as simply a consequence of one’s race, gender, or social location (Busfield, 2017; Sebring, 2021). For example, common medical gaslighting behaviors include attributing patients’ symptoms solely to stress, poor nutrition, mental health, lack of exercise, and/or weight rather than conducting more thorough examinations (Durbhakula and Fortin, 2023). Furthermore, bio-Others such as lesbian, gay, bisexual, transgender, queer, and all sexually diverse and gender diverse (LGBTQ+) individuals especially are more likely to have their symptoms dismissed without full, comprehensive examinations (Wise, 2022).

Research and data from nationwide samples in the United States (U.S.) regarding medical-related stigma experienced by LGBTQ+ people are scant. As such, the primary goals of this study were to understand LGBTQ+ patients’ healthcare experiences and elucidate pervasive systemic disparities. Specifically, this study aimed to highlight the presence of medical-related gaslighting as a form of discrimination, overt medical-related traumas, and levels of distrust in healthcare providers and systems within the United States. Through identification, documentation, and discussion of these systemic disparities, this study hoped to educate and subsequently improve the affirmation of healthcare professionals and systems in order to foster safer, more trusting patient-doctor relationships and promote LGBTQ+ health and well-being.

## Materials and methods

In March 2023, an anonymous, self-report, cross-sectional survey was distributed online via a third-party vendor to a national sample of 2,600 U.S. residents. This work was undertaken through a partnership between OutCare Health (Nowaskie, 2021) and Healthgrades to amplify the healthcare experiences of LGBTQ+ people as well as promote LGBTQ+ affirming care.

Participation was voluntary and constituted consent. Participants self-disclosed their age, sexual orientation, gender identity, race/ethnicity, education, employment, household income, relationship status, household size, parenting status, and region of residence. Participants also answered questions about their healthcare experiences. This data was provided by Healthgrades, who commissioned the survey, to the corresponding author. Because data was deidentified, this study was deemed not human subjects research by the University of Southern California Institutional Review Board (Protocol #UP-24-00660).

For simplification of reporting, the variables education, employment, household income, relationship status, household size, and parenting status were categorized into groups. Participants were categorized into two groups based on their sexual orientation and gender identity: (1) LGBTQ+ people (*N* = 1,200) and (2) exclusively cisgender and heterosexual people (*N* = 1,400). Participants were then weighted to be nationally representative of the general U.S. population based on age, gender identity, race/ethnicity, and region of residence. Frequencies of demographic and healthcare experience questions were computed. Overall frequencies of any experience of medical-related gaslighting and trauma were also computed. Odds ratios were calculated to compare healthcare experiences between LGBTQ+ people and cisgender and heterosexual people. Given the survey length, question magnitude, and focus of this manuscript, items related to medical-related gaslighting, trauma, and distrust are reported here.

Additionally, it is well known that many demographic variables, including age, race/ethnicity, education, employment, individual annual income, and state of residence, may influence health outcomes. To avoid difficult interpretations due to analytical complexity, comparative analyses reported here are based solely on sexual orientation and gender identity, i.e., LGBTQ+ people compared to cisgender and heterosexual people.

## Results

Many LGBTQ+ people (*n* = 891) and cisgender and heterosexual people (*n* = 980) expressed their perspectives and voices (Table 1). After weighting, a representative sample of LGBTQ+ people (*n* = 815) and cisgender and heterosexual people (*n* = 857) were analyzed. More than half of LGBTQ+ people were between 18 to 34 years old, White/Caucasian, had higher education, employed, earned between $25,000–$100,000 annually, lived in a household of at least three people, and did not have children; nearly half of LGBTQ+ people were partnered; and they lived across the U.S.

**Table 1:** Demographics.

	LGBTQ+ people	Cisgender and heterosexual people
Sexual orientation		
Asexual	62 (6.5%)	
Bisexual	382 (40.1%)	
Gay	62 (6.5%)	
Heterosexual/straight	155 (16.3%)	
Lesbian	86 (9.0%)	
Pansexual	95 (10.0%)	
Queer	47 (4.9%)	
Questioning	19 (2.0%)	
Not listed	7 (0.7%)	
Do not identify with terms/labels	20 (2.1%)	
Prefer not to disclose	17 (1.8%)	
Gender identity		
Agender	15 (1.6%)	
Cisgender man	89 (9.3%)	
Cisgender woman	458 (48.1%)	
Genderqueer	37 (3.9%)	
Nonbinary	73 (7.7%)	
Transgender man	20 (2.1%)	
Transgender woman	9 (0.9%)	
Not listed	91 (9.6%)	
Do not identify with terms/labels	150 (15.8%)	
Prefer not to disclose	89 (9.3%)	
Age		
18–24	131 (14.7%)	123 (12.6%)
25–34	320 (35.9%)	215 (21.9%)
35–44	230 (25.8%)	256 (26.1%)
45–54	117 (13.1%)	118 (12.0%)
55–64	67 (7.5%)	179 (18.3%)
65+	26 (2.9%)	82 (8.4%)
Unknown or prefer not to disclose		7 (0.7%)
Race/ethnicity		
American Indian/Alaska Native	3 (0.3%)	3 (0.3%)
Asian	40 (4.5%)	36 (3.7%)
Black/African American	63 (7.1%)	67 (6.8%)
Hispanic/Latino	71 (8.0%)	122 (12.4%)
White/Caucasian	669 (75.1%)	740 (75.5%)
Unknown or prefer not to disclose	45 (5.1%)	12 (1.2%)
Education		
Higher education	658 (73.8%)	723 (73.8%)
No higher education	199 (22.3%)	192 (20.0%)
Unknown or prefer not to disclose	34 (3.8%)	65 (6.6%)
Employment		
Employed	551 (61.8%)	614 (62.7%)
Not employed	307 (34.5%)	296 (30.2%)
Unknown or prefer not to disclose	33 (3.7%)	70 (7.1%)
Household income		
Under $25,000	191 (21.4%)	125 (12.8%)
$25,000–$44,999	189 (21.2%)	144 (14.7%)
$45,000–$100,000	310 (34.8%)	357 (36.4%)
$100,000+	115 (12.9%)	230 (23.5%)
Unknown or prefer not to disclose	86 (9.7%)	124 (12.7%)
Relationship status		
Partnered	419 (47.0%)	566 (57.8%)
Not partnered	426 (47.8%)	337 (34.4%)
Unknown or prefer not to disclose	46 (5.2%)	77 (7.9%)
Household size		
1	157 (17.6%)	135 (13.8%)
2	258 (29.0%)	251 (25.6%)
3+	451 (50.6%)	534 (54.5%)
Unknown or prefer not to disclose	25 (2.8%)	60 (6.1%)
Parenting status		
Have children	382 (42.9%)	594 (60.6%)
Do not have children	475 (53.3%)	311 (31.7%)
Unknown or prefer not to disclose	34 (3.8%)	75 (7.7%)
Region		
Midwest	224 (25.1%)	206 (21.0%)
Northeast	163 (18.3%)	173 (17.8%)
South	324 (36.4%)	392 (40.0%)
West	179 (20.1%)	209 (21.3%)
Unknown or prefer not to disclose	1 (0.1%)	

Overall, significantly more LGBTQ+ people reported experiencing medical-related gaslighting (46.5% vs. 26.5%, *ORs* 1.75–2.80) and trauma (18% vs. 8.9%, *ORs* 1.63–2.66) than cisgender and heterosexual people (Table 2). Nearly half of LGBTQ+ people conveyed undergoing medical-related gaslighting by their providers. Individual items showed that compared to cisgender and heterosexual people, significantly more LGBTQ+ people felt dismissed, minimized, and ignored. Likewise, nearly one-fifth of LGBTQ+ people described enduring trauma, including emotional and physical traumas, at a multitude higher rate than cisgender and heterosexual people. Additionally, significantly less LGBTQ+ people (*ORs* 0.46–0.53) reported trusting primary care providers (59.8% vs. 74.1%), medical specialists (56.5% vs. 71.7%), pharmaceutical companies (17% vs. 28%), insurance companies (15.9% vs. 29.3%), and U.S. healthcare systems (17.8% vs. 30.4%) than cisgender and heterosexual people. Slightly more than half of LGBTQ+ people disclosed that they trusted individual providers, while less than one-fifth stated that they trusted larger healthcare systems.

**Table 2:** Healthcare experiences.

	LGBTQ+ people	Cisgender and heterosexual people	OR, 95% CI, *p*-value
*Gaslighting*	379 (46.5%)	227 (26.5%)	2.41, 1.97–2.96, *p* < 0.001
I have felt dismissed or not taken seriously by a doctor when discussing my health concerns or symptoms.	238 (29.2%)	110 (12.8%)	2.80, 2.18–3.60, *p* < 0.001
Doctors have minimized or ignored symptoms I believed were significant or indicative of a health issue.	207 (25.4%)	107 (12.5%)	2.39, 1.85–3.08, *p* < 0.001
I have been made to feel like my health concerns were exaggerated or not significant by a doctor.	180 (22.1%)	100 (11.7%)	2.15, 1.64–2.80, *p* < 0.001
I have been told my symptoms were “all in my head” or otherwise psychological when I felt they were physical.	126 (15.5%)	55 (6.4%)	2.67, 1.91–3.72, *p* < 0.001
I have stopped treatment for a medical condition because a doctor did not take my symptoms or concerns seriously.	109 (13.4%)	61 (7.1%)	2.01, 1.45–2.80, *p* < 0.001
I have been discouraged from seeking a second opinion or questioning a doctor’s diagnosis or treatment plan.	75 (9.2%)	47 (5.5%)	1.75, 1.20–2.55, *p* = 0.004
*Trauma*	147 (18.0%)	76 (8.9%)	2.26, 1.68–3.04, *p* < 0.001
I have experienced medical-related emotional trauma.	109 (13.4%)	47 (5.5%)	2.66, 1.86–3.80, *p* < 0.001
I have experienced medical-related physical trauma.	50 (6.1%)	33 (3.9%)	1.63, 1.04–2.56, *p* = 0.033
I have experienced medical-related sexual trauma.	25 (3.1%)	16 (1.9%)	1.66, 0.88–3.14, *p* = 0.116
*Trust*			
Primary care providers	438 (59.8%)	601 (74.1%)	0.52, 0.42–0.64, *p* < 0.001
Medical specialists	406 (56.5%)	563 (71.7%)	0.51, 0.41–0.63, *p* < 0.001
Pharmaceutical companies	133 (17.0%)	235 (28.0%)	0.53, 0.42–0.67, *p* < 0.001
Insurance companies	125 (15.9%)	247 (29.3%)	0.46, 0.36–0.58, *p* < 0.001
U.S. healthcare systems	141 (17.8%)	256 (30.4%)	0.50, 0.39–0.63, *p* < 0.001

OR, odds ratio, CI, confidence interval. For gaslighting and trauma items, LGBTQ+ people, *n* = 815, cisgender and heterosexual people, *n* = 857. For trust items: primary care providers (LGBTQ+ people, *n* = 733, cisgender and heterosexual people, *n* = 811), medical specialists (LGBTQ+ people, *n* = 719, cisgender and heterosexual people, *n* = 785), pharmaceutical companies (LGBTQ+ people, *n* = 782, cisgender and heterosexual people, *n* = 840), insurance companies (LGBTQ+ people, *n* = 784, cisgender and heterosexual people, *n* = 844), and U.S. healthcare systems (LGBTQ+ people, *n* = 791, cisgender and heterosexual people, *n* = 842).

## Discussion

The goal of this study was to examine medical-related gaslighting, trauma, and distrust among LGBTQ+ people. Overall, compared to cisgender and heterosexual people, LGBTQ+ people reported higher rates of medical-related gaslighting and trauma. Likewise, LGBTQ+ people conveyed less trust in healthcare providers and systems. These findings mirror existing literature that demonstrates that discrimination is widely experienced by LGBTQ+ people within healthcare (Casey et al., 2019).

Since the institutionalization—and subsequent power differentials—of healthcare, communities composed of marginalized identities have experienced increasing levels of stigma, discrimination, and iatrogenesis (Atuk, 2024). Iatrogenesis is chronic and pervasive harm, often originating from individual healthcare providers and staff themselves as well as systems at large. Individuals who identify as LGBTQ+ have historically had polarizing, iatrogenic relationships within healthcare. Gaslighting is one of many forms of ubiquitous medical-related harm and violence experienced by LGBTQ+ people. For example, during the initial discovery of human immunodeficiency virus (HIV)/acquired immunodeficiency syndrome (AIDS) in the 1980s, the disease was originally and commonly referred to as “Gay-Related Immune Deficiency” (Ayala and Spieldenner, 2021). This label fostered a false correlation between sexually diverse people and the virus, which led to discrimination and neglect as individuals with HIV/AIDS struggled to find medical care and faced medical gaslighting and victimization at the hands of medical professionals. Today, LGBTQ+ individuals often face significant health disparities such as increased rates of sexually transmitted infections, mental health conditions, substance use, and suicide (Lund and Burgess, 2021). These health disparities are strongly associated with direct and indirect consequences of oppression, prejudice, discrimination, and violence that LGBTQ+ people often face. This phenomenon that sociological aspects of a person’s life and experiences are directly linked to one’s health outcomes was eventually conceptualized and popularized in the minority stress model (Meyer, 2003). Minority stress refers to the additional stressors that individuals encounter as a direct result of their identification and association within marginalized groups (Lund and Burgess, 2021; Meyer, 2003). The model outlines two categories of minority stress: proximal stressors and distal stressors. Proximal stressors refer to internal experiences and stress related to self-concealment of sexual orientation, such as internalized homophobia. On the other hand, distal stressors refer to external negative experiences related to sexual orientation and gender identity including harassment, violence, and discrimination. Both types of processes lead to chronic stress, which over time can manifest as physical and mental health conditions (Meyer, 2003).

Utilizing a minority stress framework (Meyer, 2003), distal stressors such as gaslighting and trauma by healthcare providers and systems likely induces proximal stressors such as internal stress, poor self-esteem, and distrust within LGBTQ+ patients. Due to substantial lack of LGBTQ+ awareness, education, and training (Nowaskie, 2021), cissexism, cisgenderism, cisnormativity, and heterosexism are promoted throughout entire healthcare journeys, from start to finish. Many healthcare providers and systems do not obtain sexual orientation and gender identity information nor screen for or treat unique LGBTQ+ health risks and disparities (Lund and Burgess, 2021). Likewise, similar to the disparities of trauma documented in this study, overt violence from healthcare providers and staff can occur, as gender diverse people are four times more likely than cisgender people to experience victimization (Flores et al., 2021). Additionally, nearly one-third of gender diverse people report enduring at least one negative experience from their healthcare provider, such as verbal harassment or refusal of treatment, because of their identity. Moreover, these discriminatory forces continue to produce, perpetuate, and exacerbate health disparities (Ramos, 2021). These pervasive, vicious cycles of medical-related discrimination may then lead many LGBTQ+ people to avoid healthcare altogether due to anticipated stigma, fear, and distrust in providers and systems (Bullock, 2023; Casey et al., 2019; James et al., 2016). For example, due to verbal and physical harassment within healthcare, LGBTQ+ people may forego their care and obtain unregulated or even illegal forms of care such as hormones and surgical procedures (Chong et al., 2021). Because of this overt discrimination, it is not surprising that in this study, only half of LGBTQ+ people reported trusting providers and less than one-fifth conveyed trusting healthcare systems.

While this data offers cross-sectional insight into the current state of medical-related gaslighting, trauma, and distrust, it is descriptive of a point in time of the current stigma from and within particular contexts (i.e., providers and systems). Healthcare journeys involve many more staff and contexts than this data examined. Consequently, further considerations of medical-related gaslighting, trauma, and distrust from various healthcare professionals and staff and within specific teams, groups, departments, and communities are necessary. Additionally, this data does not account for the impact of social and political discriminations within healthcare. For example, past and current socio-politico-medico systemic and structural marginalizations, such as anti-LGBTQ+ proposals and laws, likely worsen medical-related gaslighting, trauma, and distrust and prevent their alleviation. Explorations into these complexities are paramount and will likely yield solutions-focused approaches for progress and affirmation.

While insufficient education, training, and affirmation among healthcare providers and systems are likely fundamental barriers to alleviating medical-related gaslighting, trauma, and distrust, such shortcomings can be targeted by advocacy, online resources, and organizations. For example, OutCare Health, an international nonprofit LGBTQ+ health equity organization, promotes affirming healthcare by educating current and future healthcare providers and systems (Nowaskie, 2021; Nowaskie and Garrison, 2024). Through multidisciplinary and multidimensional approaches, OutCare Health supports LGBTQ+ and healthcare communities worldwide with comprehensive information, resources, support, and education (Nowaskie, 2021; Nowaskie and Garrison, 2024; Nowaskie et al., 2024; Patel and Nowaskie, 2023).

### Limitations

There were notable study limitations. Although many demographic variables were collected in the survey, comparisons related to intersectionality were not undertaken. Rather, while acknowledging the multitude of diversity within LGBTQ+ populations, these data nonetheless present a homogenized perspective. Given the amount of data collected, it is appreciated that many various analyses incorporating several demographic variables could have been undertaken, reported, and interpreted in a myriad of ways, including investigations across specific sexual orientations and gender identities. Further comparative explorations, e.g., with LGBTQ+ subgroups such as gender diverse identities and LGBTQ+ people of color, are strongly encouraged to understand the similarities and differences in healthcare experiences among these groups.

## Conclusion

Medical-related gaslighting, trauma, and distrust are pervasive systemic disparities among LGBTQ+ people. These forms of discrimination create significant barriers for equitable care for LGBTQ+ communities. Addressing these challenges will require ongoing, lifelong motivation, dedication, and commitment for LGBTQ+ education, advocacy, and leadership to dismantle current prejudiced practices and foster more inclusive, supportive, affirming healthcare environments.

## Data Availability

The original contributions presented in the study are included in the article/supplementary material, further inquiries can be directed to the corresponding author.
